# Management of six episodes of intraoperative cardiac arrests in an infant with traumatic bronchial rupture: A case report

**DOI:** 10.1097/MD.0000000000037891

**Published:** 2024-04-19

**Authors:** Haikou Yang, Jixiang Zhu, Jian Sun

**Affiliations:** a Department of Anesthesiology, Affiliated Hospital of Yang Zhou University Medical College, Huai’an Maternal and Child Health Care Center, Huai’an, Jiangsu, People’s Republic of China; b Department of Anesthesiology, Yancheng First Hospital, Affiliated Hospital of Nanjing University Medical School, Yancheng, Jiangsu, People’s Republic of China.

**Keywords:** cardiac arrest, case report, hypoxemia, infant, traumatic bronchial rupture

## Abstract

**Rationale:**

Traumatic bronchial rupture in infants usually necessitates surgical intervention, with few reports documenting instances of multiple cardiac arrests occurring during surgery under conditions of severe hypoxemia.

**Patient concerns:**

A 3-year-old boy after trauma presented with severe hypoxemia for 2 days and was urgently transferred to the operating room for surgery, 6 episodes of cardiac arrest happend during surgery.

**Diagnoses:**

The baby was diagnosed with bronchial rupture based on the history of trauma, clinica manifestations, and intraoperative findings.

**Interventions:**

Intrathoracic cardiac compression and intravenous adrenaline were administrated.

**Outcomes:**

The normal sinus rhythm of the heart was successfully restored within 1 minute on each occasion, facilitating the smooth completion of the surgical procedure. By the end of surgery, SpO_2_ levels had rebounded to 95% and remained stable.

**Lessons:**

Inadequate management of bronchial ruptures in infants frequently coincides with severe hypoxemia, necessitating immediate surgical intervention. Prompt identification and management of cardiac arrest by anesthetists during surgery is imperative to reduce mortality.

## 1. Introduction

Bronchial rupture is uncommon in infants within clinical practice. Airway impairment results in air leakage, which hampers respiratory function and leads to the development of acute severe dyspnea, hypoxia, and CO_2_ retention. Emergency surgical intervention is necessary if dyspnea cannot be alleviated with closed thoracic drainage. The present report focuses on the anesthesia management process for emergency surgery in a pediatric patient with severe hypoxemia resulting from a delayed diagnosis of bronchial rupture that involved several cardiac arrests during surgery.

## 2. Case report

**Figure 1.**
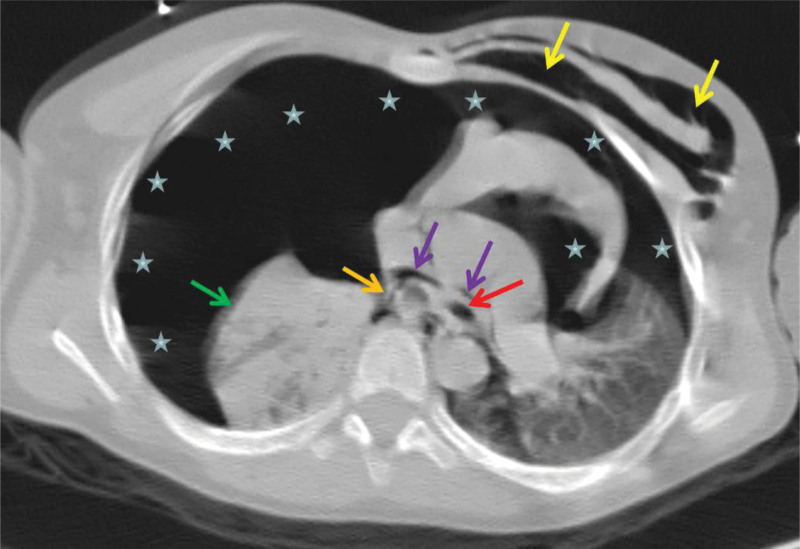
The image of CT scan. The left main bronchus demonstrated adequate gas filling and intact wall integrity (as indicated by the red arrow). Conversely, the right main bronchus exhibited collapse with poor wall continuity (as shown by the orange arrow). There was associated right lung collapse and atelectasis (as highlighted by the green arrow). A substantial pneumothorax was observed on the right side, extending across the mediastinum anteriorly into the left chest cavity (as depicted by the stars). Mediastinal air accumulation was evident (as denoted by the purple arrow), collectively suggesting a rupture in the right bronchus. Notably, there was air entrapment along the left chest wall in this image (indicated by the yellow arrow). CT = computed tomography.

A 3-year-old boy experienced dyspnea following a fall from a height of approximately 8 m on a hard floor. Tracheal intubation and bilateral closed thoracic drainage were performed after injury. Two days later, he was transferred to our hospital because inability to maintain normal SpO_2_ levels, resulting in a significant drop of approximately 30%. When entering the operating room, the patient blood pressure was 50/18 mm Hg, heart rate was 122 beats/min, SpO_2_ was approximately 20%, Glasgow Coma Scale score was 6 points, and bilateral pupillary light reflexes were sluggish, with a diameter of 3 mm. Tracheal intubation was performed with assisted breathing using a bag and extensive subcutaneous emphysema was observed in the neck and chest. Bilateral thoracic drainage tubes were inserted to evacuate the hemorrhagic fluid, and a significant amount of gas leaked from the right chest cavity, whereas the left tube drained only hemorrhagic fluid (Video, http://links.lww.com/MD/M241). Respiratory auscultation revealed bilateral decreased breath sounds, with scattered dry and moist crackles. Respiratory sounds were absent from the upper lobe of the right lung. The results of the blood gas analysis indicated a pH level of 7.15, pressure of carbon dioxide of 85.3 mm Hg, PO_2_ of 31.1 mm Hg, and hemoglobin concentration of 96 g/L. Computed tomography revealed adequate gas filling and an intact wall in the left main bronchus, whereas the right main bronchus exhibited collapse with poor wall continuity. Additionally, the right lung exhibited collapse and atelectasis accompanied by a substantial pneumothorax on the right side that extended into the left chest anterior to the mediastinum. Furthermore, extensive air accumulation is observed in the mediastinum. Overall, rupture of the right bronchus was observed. In addition, the left chest wall showed air accumulation (Fig. 1). Fiberoptic bronchoscopy revealed localized edema and hemorrhagic secretions in the right main bronchus during emergencies. After opening the thoracic cavity, a fiberoptic bronchoscope was used to precisely locate the site of rupture under illumination. During the surgery, the surgical field of the right bronchial tube was transparent, and the location of the rupture was determined. An endobronchial blocker was inserted during the surgery with intermittent single-lung ventilation under the guidance of the surgeon. The surgical procedures performed included right middle and lower lobectomy as well as upper lobe bronchial reconstruction. Anesthesia management primarily involved sevoflurane inhalation, with intermittent injections of fentanyl and vecuronium.

Unfortunately, 6 cardiac arrests occurred during the operation. Intrathoracic cardiac compression, intravenous administration of atropine, and repeated intravenous injections of epinephrine facilitated return to spontaneous circulation (ROSC). Fortunately, the heart successfully reverted to its normal rhythm within 1 minute each time. Simultaneously, intravenous administration of dopamine was employed to maintain normal blood pressure, while sodium bicarbonate and calcium gluconate were used to correct acid-base imbalance and electrolyte disorders. Additionally, therapeutic head-cooling measures were implemented. Postoperative blood gas analysis revealed the following: pH 7.20, pressure of carbon dioxide 58.1 mm Hg, PO_2_ 60.8 mm Hg, and hemoglobin concentration 92 g/L. Following the completion of the surgery, SpO_2_ rebounded to approximately 95% and remained stable. Subsequently, the infant was transferred to the intensive care unit via tracheal catheter. Regrettably, despite these favorable advancements, his guardians decided to terminate the treatment due to concerns regarding potential neurological complications. The guardian consented to publication of this case report.

## 3. Discussion

The developmental features of language and psychology make it challenging for young children to provide accurate descriptions and cooperate in examinations following trauma. Moreover, the frequent occurrence of complex injuries such as traumatic brain injury, abdominal organ injury, and limb fractures in pediatric patients often leads to overlooked or delayed diagnosis in children.^[1]^

Once diagnosed, closed thoracic drainage should be promptly implemented, and surgical intervention should be actively pursued to rectify airway leakage.^[2]^ Due to delayed diagnosis before surgery, despite tracheal intubation and closed thoracic drainage, airway leakage did not change, resulting in severe hypoxemia following significant ventilation decline and subsequent pathological cascades. The mortality rate of emergency operations in patients with traumatic bronchial rupture was 11.8%. The primary causes of fatality were attributed to cardiac arrest and cerebral hypoxia^[3]^; factors that affect the prognosis of resuscitation include age, American Society of Anesthesiologists grade, comorbidities, and type of surgery.^[4]^

The detection of cardiac arrest can be facilitated by continuous monitoring of the electrocardiogram and SPO_2_, as well as by direct visualization of the heartbeat during this surgical procedure. Immediate administration of atropine and epinephrine and intrathoracic cardiac compression is imperative. With regard to the occurrence of 6 cardiac arrests during surgery, 3 main aspects have been analyzed and considered for treatment. First, the integrity of the trachea disappears following traumatic tracheal rupture, posing challenges for airway management. Blind tracheal intubation through a distal tracheal rupture poses a significant risk as it may lead to complete airway obstruction and potentially exacerbate tracheal injury by converting an incomplete rupture into a full rupture or causing retraction of the fractured distal trachea into the thoracic cavity.

In this case, a fiberoptic bronchoscope was employed to facilitate the localization of the rupture site, and with surgical assistance, a bronchial blocker was successfully inserted. There was a significant reversal of hypoxemia and carbon dioxide retention following bronchial reconstruction. Enhancement of ventilation is a prerequisite for reversing severe hypoxemia in children, as it effectively corrects acid-base and electrolyte imbalances within the body, thereby improving vital organ functions, such as the heart, brain, and kidneys and promoting children survival. Second, the patient presented with inadequate tidal volume, severe hypoxemia, acidosis, and compensatory hyperkalemia. These pathological factors predispose the patient to myocardial hypoxia, thereby augmenting susceptibility to arrhythmias or even cardiac arrest during surgery which constitutes the primary etiology of cardiac arrest. In addition to maintaining a moderate depth of anesthesia during the operation, dopamine was administered to maintain blood pressure. To address preexisting internal environmental disorders, we cautiously utilized sodium bicarbonate and calcium supplementation. Notably, these measures must be gradually implemented throughout the procedure. Finally, a dense network of nerves terminates in the tracheal wall, lung hilum, pleura, and the heart. This anatomical arrangement renders patients vulnerable to inhibitory neuronal reflexes during surgical exploration, potentially resulting in cardiac arrest. Therefore, local anesthetics were administered to inhibit this pathway after the first cardiac arrest. The prompt return of spontaneous circulation is crucial because effective cardiac compressions during cardiac arrest can only generate a normal cardiac output of approximately 30%.^[5]^ In addition to the immediate cessation of surgical and intrathoracic cardiac compressions, the administration of intravenous epinephrine was also imperative after cardiac arrest. Adrenaline is the most significant drug for cardiac arrest as it enhances cardiac autonomy, induces vasoconstriction, and elevates aortic diastolic pressure and coronary perfusion pressure, thereby augmenting the likelihood of achieving ROSC.^[6]^ Some studies have suggested that intracoronary adrenaline may have a beneficial effect on ROSC and improve long-term survival and neurological prognosis.^[7]^ Close collaboration among the members of the surgical team is crucial.

The most significant regret in this case was the protracted delay in diagnosis, which led to severe disruption of the body homeostasis. At this stage, a cascade of catastrophic consequences resulting from inadequate ventilation has already taken hold, inevitably causing damage to the vital cardiovascular and nervous systems. Infants may have benefited from extracorporeal membrane oxygenation-assisted circulation, if feasible, to improve severe hypoxia and potentially facilitate a safe perioperative course. Additionally, it can serve as a bridge for subsequent treatment and the enhancement of patient survival.^[8]^

## 4. Conclusions

We successfully resuscitated a severely hypoxemic pediatric patient who experienced 6 intraoperative cardiac arrests during surgery, and we were able to restore airway integrity, thereby increasing tidal volume. Although this is a rare case of emergency surgery in young children, it presents significant challenges for both anesthesiologists and surgeons.

## Author contributions

**Data curation:** Jian Sun.

**Investigation:** Haikou Yang.

**Methodology:** Haikou Yang, Jixiang Zhu.

**Project administration:** Jian Sun.

**Supervision:** Jian Sun.**Visualization:** Jixiang Zhu.

**Writing – original draft:** Haikou Yang.

**Writing – review & editing:** Haikou Yang, Jixiang Zhu.

## Supplementary Material


